# Association between ischemic heart disease and colorectal neoplasm: a systematic review and meta-analysis

**DOI:** 10.1186/s40064-016-3349-0

**Published:** 2016-09-27

**Authors:** Yoo Jee Hee, Chang Seok Bang, Gwang Ho Baik, In Soo Shin, Ki Tae Suk, Tae Young Park, Dong Joon Kim

**Affiliations:** 1 Department of Internal Medicine, Chuncheon Sacred Heart Hospital, Hallym University College of Medicine, 153, Gyo-dong, Chuncheon, Gangwon-do 200-704 South Korea; 2 College of Education, Jeonju University, Jeonju, South Korea

**Keywords:** Colorectal neoplasm, Ischemic heart disease, Coronary artery disease, Meta-analysis

## Abstract

**Purpose:**

Colorectal neoplasm and ischemic heart disease (IHD) share common risk factors. However, clinical guidance about screening or surveillance of colorectal neoplasm in patients with IHD has not been made. The aim of this study was to investigate the relationship between IHD and the development of colorectal neoplasm.

**Methods:**

A systematic literature review was conducted using the core databases (MEDLINE through PubMed, EMBASE, and the Cochrane Library). The data about the association between IHD and the development of colorectal neoplasm were extracted and analyzed using odds ratio (OR). A random effect model was applied. The methodological quality of the enrolled studies was assessed by the Newcastle–Ottawa Scale. Publication bias was evaluated through the funnel plot with trim and fill method, Egger’s test, and the rank correlation test.

**Results:**

A total of 3069 patients from 4 non-randomized studies were enrolled. IHD was associated with colorectal neoplasm (OR 1.869, 95 % CI 1.375–2.542, *p* < 0.001). Sensitivity analyses showed consistent results. Publication bias was not detected.

**Conclusion:**

Patients with IHD is associated with colorectal neoplasm, which warrants screening or surveillance of colorectal neoplasm in this group of patients.

## Background

Colorectal cancer and ischemic heart disease (IHD) are one of the leading causes of death worldwide. Colorectal cancer is the third most common cause of mortality due to cancer (Begg and Mazumdar 1994). IHD is responsible for about one-third of mortality over age 35 (Borenstein et al. 2009). There have been reports about common risk factors shared by these two conditions, such as diabetes, smoking, dyslipidemia, or sedentary life style (including alcohol and smoking) (Botteri et al. 2008; Cai et al. 2015; Chan et al. 2006; Chan et al. 2007; Crosara Teixeira et al. 2014). Among the various risk factors, obesity and visceral fat accumulation are getting attention with related conditions including cardiovascular, metabolic, and cancerous diseases (Deeks et al. 2003). Insulin resistance and chronic inflammation by obesity and visceral fat accumulation have been assumed as a mechanism for the development of colorectal neoplasm (including colorectal cancer and its precursor adenoma) and IHD (DerSimonian and Laird 1986; Duval and Tweedie 2000). Although many epidemiologic studies related to this topic, clinical guidance about screening or surveillance of colorectal neoplasm in patients with IHD has not been made. The aim of this study was to investigate the relationship between IHD and development of colorectal neoplasm.

## Methods

### Literature search

MEDLINE (through PubMed), EMBASE, and the Cochrane Central Register of Controlled Trials (CENTRAL) in the Cochrane Library were searched using common keywords related to colorectal neoplasm and IHD (from inception to December 2015). Medical Subject Headings (MeSH) were used because all 3 databases permit searches using MeSH terminology. The keywords included ‘ischemic heart disease’, ‘coronary artery disease’, ‘colorectal neoplasm’, ‘colorectal cancer’, and ‘colorectal tumor’ using Boolean operators. Only publications on human subjects were sought, and the bibliographies of relevant articles were also reviewed to identify additional studies. The language of the publications was not restricted.

### Selection criteria

We included randomized or non-randomized studies that met the following criteria: 1. the study was designed to evaluate the prevalence of IHD in patients with colorectal neoplasm or vice versa, or 2. the study was designed to evaluate the association between IHD and colorectal neoplasm. The exclusion criteria were as follows: (1) incomplete data, (2) review article, or (3) abstract-only study.

### Selection of relevant studies

Two of the authors (C.S.B. and G.H.B.) independently evaluated the eligibility of all studies retrieved from the databases based on the predetermined selection criteria. The abstracts of all identified studies were reviewed to exclude irrelevant articles. Full-text reviews were performed to determine whether the inclusion criteria were satisfied by the remaining studies. Disagreements between the two evaluators were resolved by discussion or by consultation with a third author (D.J.K.).

### Methodological quality

The methodological quality of the enrolled studies was assessed using the Newcastle–Ottawa Scale (NOS) for non-randomized studies. The NOS is categorized into three parameters: the selection of the study population, the comparability of the groups, and the ascertainment of the exposure or outcome. Each parameter consists of subcategorized questions: selection (*N* = 4), comparability (*N* = 1), and exposure or outcome (*N* = 3) (Egger et al. 1997; Ellenberg 1994). Stars awarded for each item serve as a quick visual assessment for the methodological quality of the studies. A study can be awarded a maximum of nine stars, which indicates the highest quality. Two of the authors (C.S.B. and G.H.B.) independently evaluated the methodological quality of all studies, and any disagreements between the two evaluators were resolved by discussion or by consultation with a third author (D.J.K.). Sensitivity analyses were performed.

## Statistics

Comprehensive Meta-Analysis (CMA) software (version 2.2.064, Biostat; Borenstein M, Hedges L, Higgins J and Rothstein H. Englewood, NJ, USA) was used for this meta-analysis. We calculated the odds ratios (ORs) with 95 % confidence intervals (CIs) using 2 × 2 tables whenever possible from the original articles in order to calculate the association between IHD and colorectal neoplasm. Heterogeneity was determined using the *I*^2^ test developed by Higgins, which measures the percentage of total variation across studies (Frishman et al. 2001). *I*^2^ was calculated as follows: *I*^2^ (%) = 100 × (Q − df)/Q, where Q is Cochrane’s heterogeneity statistic and df signifies the degree of freedom. Negative values for *I*^2^ were set to zero, and an *I*^2^ value over 50 % was considered to be of substantial heterogeneity (range 0–100 %) (Hedges and Olkin 1985). Pooled-effect sizes with 95 % CIs were calculated using a random effects model and the method of DerSimonian and Laird because of methodological heterogeneity (Higgins and Thompson 2002). These results were confirmed by the *I*^2^ test. A fixed effects model using the inverse variance-weighted (Woolf’s) method was used in the sensitivity analyses, including cumulative and one-study-removed analyses, based on the assumption of a common effect size shared by the studies within each subgroup (Higgins et al. 2003; Hong et al. 2010). Significance was set at *p* = 0.05 in both models. Publication bias was evaluated using Begg’s funnel plot, Egger’s test of the intercept, Duval and Tweedie’s trim and fill, and Begg and Mazumdar’s rank correlation test (Larsson et al. 2005; Lloyd-Jones et al. 2010; Neugut et al. 1998; Siegel et al. 2012; Stang 2010).

## Results

### Identification of relevant studies

**Fig. 1:**
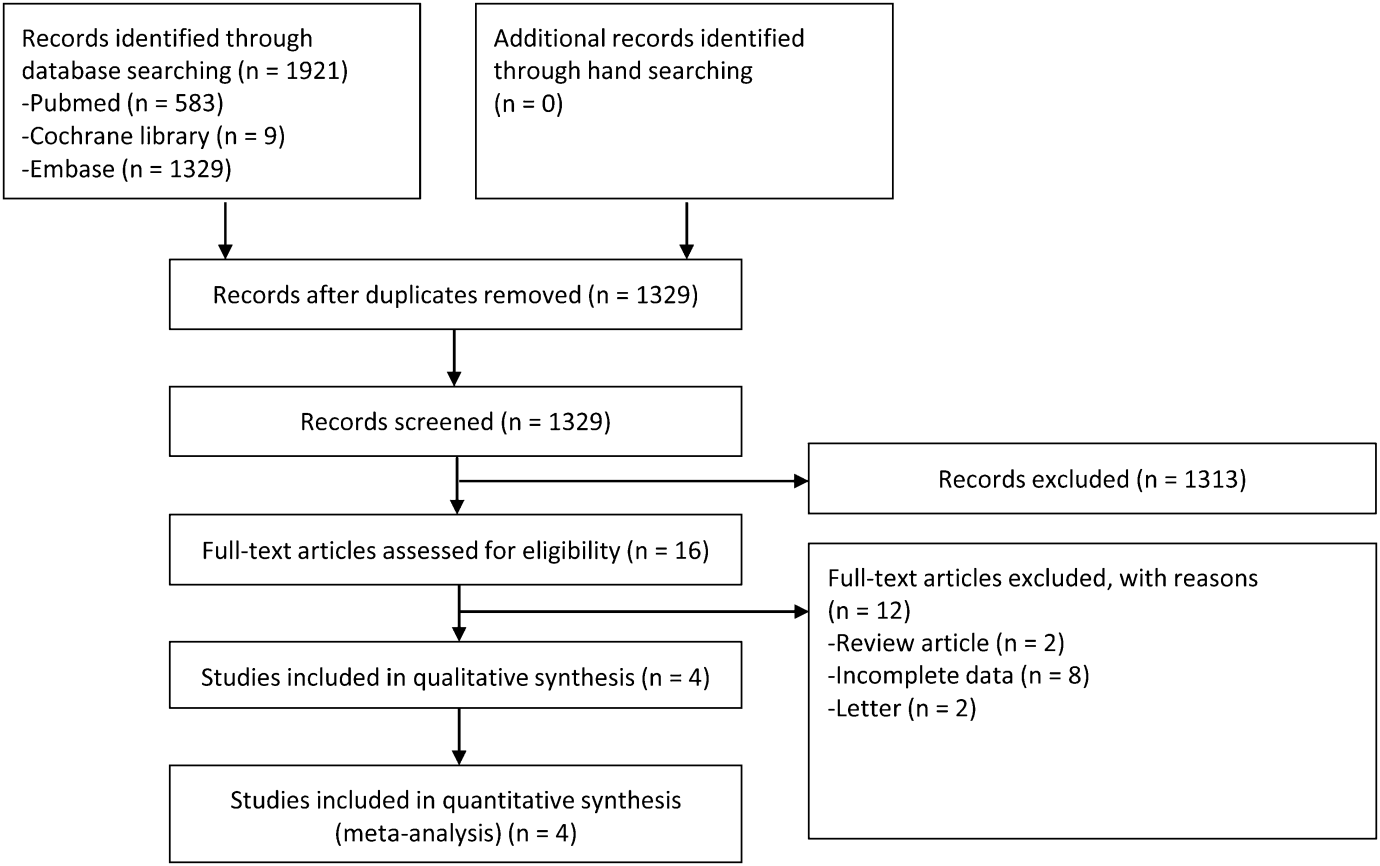
Flow diagram for identification of relevant studies

Figure 1 illustrates a flow diagram of how relevant studies were identified. A total of 1921 articles was identified by a search of 3 core databases. In all, 592 duplicate studies and an additional 1313 studies were excluded during the initial screening through a review of the titles and abstracts. The full texts of the remaining 16 studies were then thoroughly reviewed. Among these studies, 12 articles were excluded from the final analysis. The reasons for study exclusion during the final review were as follows: review article (*N* = 2), incomplete data (*N* = 8), and letter (*N* = 2). The remaining 4 studies (4 non-randomized studies) were included in the final analysis.

### Characteristics of studies

Within the 4 studies (Crosara Teixeira et al. 2014; DerSimonian and Laird 1986; Sterne and Egger 2001; Stone et al. 2014), we identified a total of 3069 patients (including 914 colorectal neoplasm cases and 546 IHD cases). The clinical characteristics of the patients in the included studies are shown in Table 1. The included studies were published between 1998 and 2010. All the enrolled studies were performed as non-randomized manner. Only 1 study (DerSimonian and Laird 1986) adopted prospective observational setting and remaining other studies (Crosara Teixeira et al. 2014; Sterne and Egger 2001; Stone et al. 2014) were performed in a retrospective manner. Three studies (Crosara Teixeira et al. 2014; DerSimonian and Laird 1986; Stone et al. 2014) were conducted in Asia and 1 study (Sterne and Egger 2001) was conducted in the US. Three studies (Crosara Teixeira et al. 2014; Sterne and Egger 2001; Stone et al. 2014) were conducted as a single center setting, whereas only 1 study (DerSimonian and Laird 1986) was conducted as a multicenter setting. All the included studies were written in English.

**Table 1:** Clinical data of included studies

Study	OR (95 % CI)	Study design	Patients	Adjust	Cancer or neoplasm	Country
Neugut et al. (1998)	1.18 (0.73–1.90)	Hospital-based case–control study	256 patients with colorectal cancer (18.8 % prior CAD), 322 controls (14 % prior CAD)	Age, gender	Cancer	US
Chan et al. (2006)	2.12 (1.5–3.0)	Hospital-based case–control study	1382 (373 patients with colorectal neoplasms, 168 patients with CAD)	Age, gender	Neoplasm	Hong Kong
Chan et al. (2007)	2.51 (1.43–4.35)	Hospital-based prospective observational study	621 (68 patients with advanced colonic lesions, 206 patients with CAD)	Age, gender	Advanced colonic lesion^a^	Hong Kong, China
Yang et al. (2010)	1.96 (1.15–3.35)	Cross-sectional study	488 men (217 patients with adenoma, 79 patients with significant CAD)	Age, smoking, metabolic syndrome	Adenoma	Korea

*OR* odds ratio

^a^Advanced colonic lesion: cancer or adenoma with villous component, with high-grade dysplasia, or 1 cm or larger

The age of enrolled patients ranged from 55.8 to 71.6 years (mean) distributed according to each of the study and all the enrolled studies adjusted age for the analysis of the association between IHD and colorectal neoplasm.

Only 1 study (Sterne and Egger 2001) included solely colorectal cancer cases and the remaining studies included colorectal neoplasm (Crosara Teixeira et al. 2014), advanced colonic lesion (cancer or adenoma with villous component, with high-grade dysplasia, or 1 cm or larger) (DerSimonian and Laird 1986), or colorectal adenoma cases (Stone et al. 2014).

The definition of IHD was made by reviewing of medical records in 2 studies (Crosara Teixeira et al. 2014; Sterne and Egger 2001), and by CT angiography in 1 study (Stone et al. 2014). Only 1 study used coronary angiography for the diagnosis of IHD (DerSimonian and Laird 1986).

### Association between IHD and colorectal neoplasm

**Fig. 2:**
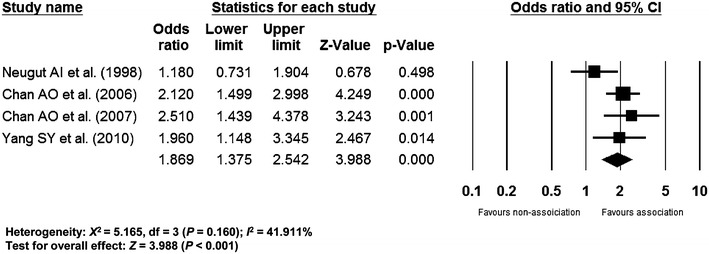
Pooled association of IHD and colorectal neoplasm. *IHD* ischemic heart disease. The size of each *square* is proportional to the study’s weight. *Diamond* is the summary estimate from the pooled studies (random effect model)

The pooled association between IHD and colorectal neoplasm showed an OR of 1.869 (95 % confidence interval (CI) 1.375–2.542, *p* < 0.001) in a random effect model-based meta-analysis of 4 studies (Fig. 2). According to the methodological quality assessment of the enrolled studies, the mean value of the awarded star was 7 [6 (1 study), 7 (2 studies), and 8 (1 study)] (Table 2). Because enrolled studies showed similar methodological quality, subgroup analysis divided by the methodological quality was not performed.

**Table 2:** Methodological quality of included studies measured by Newcastle–Ottawa Scale

Study	Selection	Comparability	Exposure or outcome	Total
Neugut et al. (1998)	☆☆☆☆	☆☆	☆	7
Chan et al. (2006)	☆☆☆☆	☆	☆☆	7
Chan et al. (2007)	☆☆☆	☆	☆☆	6
Yang et al. (2010)	☆☆☆	☆☆	☆☆☆	8

### Publication bias

**Fig. 3:**
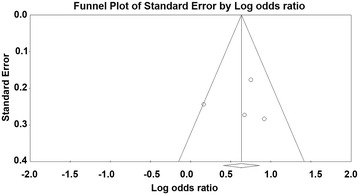
Funnel plot for publication bias. Funnel plot of studies. The *line in center* is the natural logarithm of pooled OR, and 2 oblique lines are pseudo 95 % CI. *OR* odds ratio

A funnel plot for the included studies is illustrated in Fig. 3. This plot shows a symmetrical shape. In the publication bias analysis, Egger’s regression test revealed that the intercept was −0.63 [95 % CI −18.17 to 16.91, *t*-value: 0.15, df: 2, *p* = 0.45 (1-tailed) and *p* = 0.89 (2-tailed)]. A trim and fill analysis showed that no study was missed or trimmed. The rank correlation test indicated a Kendall’s tau of 0.17 with a continuity correction [*p* = 0.37 (1-tailed) and *p* = 0.73 (2-tailed)].

Overall, there was no evidence of publication bias in this analysis.

### Sensitivity meta-analysis

**Fig. 4:**
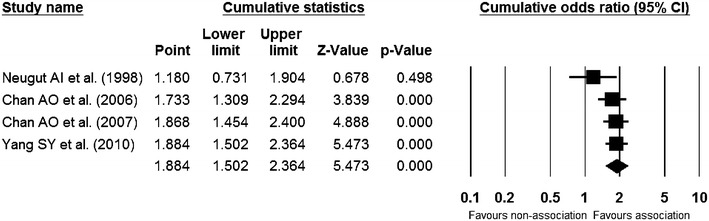
Cumulative meta-analysis of enrolled studies. *Diamond* is the summary estimate from the pooled studies (Fixed effect model)

**Fig. 5:**
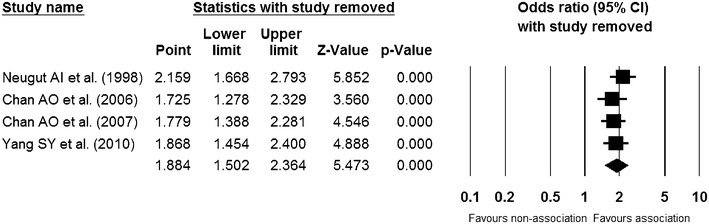
One study removed meta-analysis of enrolled studies. *Diamond* is the summary estimate from the pooled studies (Fixed effect model)

The cumulative meta-analysis of the enrolled studies (Fig. 4) and the one-study-removed meta-analysis of the included studies (Fig. 5) in the order of the year published showed a consistent result and showed no specific outlier.

## Discussion

According to this meta-analysis, patients with IHD showed significant association with colorectal neoplasm. Chronic inflammation by obesity and visceral fat accumulation have been hypothesized as the mechanism responsible for both of colorectal neoplasm and IHD, although inflammation is considered as minor pathophysiology in the development of colorectal neoplasm (Begg and Mazumdar 1994; DerSimonian and Laird 1986; Duval and Tweedie 2000).

Although the exact reason of close association with IHD and colorectal neoplasm could not be assessed in this analysis, interesting association in the prevention and treatment of both diseases exists. Aspirin is believed to have the primary preventive role in colorectal neoplasm, although the optimal dose or duration was not determined (Sutton et al. 2000). It is mainly used in the prevention of cardiovascular diseases and also for the treatment purpose. The recent meta-analysis reported that statin use before or after cancer diagnosis is related to reductions in overall and cancer-specific mortality in colorectal cancer survivors (Tiong and Brieger 2005). Statin is also recommended for the primary and secondary prevention of cardiovascular diseases (Yang et al. 2010). Although the anti-inflammatory effect of these drugs solely cannot explain the cardiovascular protective or cancer preventive effect, these associations do not seem to be just coincidence.

This study is the first meta-analysis of the association between IHD and colorectal neoplasm. The strength of this study is the rigorous literature search, although there is a lack of data. When possible, potential modifiers were detected within the articles, and sensitivity analyses were performed to confirm the robustness of the results. However, due to the paucity of enrolled studies, subgroup analysis was not made.

Despite the strengths, there are several limitations in the present study. First of all, substantial methodological heterogeneity was observed among the included studies, which had a potential effect on the pooled estimates. The most noticeable modifier was the study design. Two retrospective hospital-based case control studies, 1 hospital-based prospective observational study, and 1 cross-sectional study was enrolled (Table 1). Besides this methodological heterogeneity, non-randomized study tends to exaggerate the effects of an intervention, and this type of study is known to have an inherent bias (Zheng et al. 2011). This study included only non-randomized studies due to the lack of randomized studies relevant to this topic. This could be the cause of significant heterogeneity, although sensitivity analyses revealed consistent results which were no different from those of the main analysis. Second, the diversity of the enrolled population could be biased. Only 1 study included solely colorectal cancer, and other remaining studies included colorectal neoplasm, advanced colonic lesion, or colorectal adenoma (Table 1). Other sources of heterogeneity could be the inconsistent definition of IHD. Only 1 study used coronary angiography, and remaining studies used medical record review in 2 studies and CT angiography in 1 study for the detection of IHD. Although the *I*^2^ test showed no heterogeneity, and sensitivity analyses demonstrated consistent results, these factors could not be totally controlled for in this analysis.

The limitations described above could be a cause of heterogeneity and bias. Due to the lack of prospective or randomized studies on this topic, large-scale, well-organized, long-term follow-up studies are needed to confirm this finding. Studies revealing common molecular pathophysiologic mechanism might be preferred to confirm this result.

 In conclusion, patients with IHD is associated with colorectal neoplasm, which warrants screening or surveillance of colorectal neoplasm in this group of patients.

## Abbreviations

IHD: ischemic heart disease
MeSH: medical subject headings
OR: odds ratio
CI: confidence intervals
NOS: Newcastle–Ottawa Scale

## References

[CR1] Begg CB, Mazumdar M (1994). Operating characteristics of a rank correlation test for publication bias. Biometrics.

[CR2] Borenstein M, Hedges LV, Higgins JP, Rothstein HR (2009). Introduction to meta-analysis.

[CR3] Botteri E, Iodice S, Bagnardi V, Raimondi S, Lowenfels AB, Maisonneuve P (2008). Smoking and colorectal cancer: a meta-analysis. JAMA.

[CR4] Cai H, Zhang G, Wang Z, Luo Z, Zhou X (2015). Relationship between the use of statins and patient survival in colorectal cancer: a systematic review and meta-analysis. PLoS One.

[CR5] Chan AO, Lam KF, Tong T, Siu DC, Jim MH, Hui WM, Lai KC, Yuen MF, Lam SK, Wong BC (2006). Coexistence between colorectal cancer/adenoma and coronary artery disease: results from 1382 patients. Aliment Pharmacol Ther.

[CR6] Chan AO, Jim MH, Lam KF (2007). Prevalence of colorectal neoplasm among patients with newly diagnosed coronary artery disease. JAMA.

[CR7] Crosara Teixeira M, Braghiroli MI, Sabbaga J, Hoff PM (2014). Primary prevention of colorectal cancer: Myth or reality?. World J Gastroenterol.

[CR8] Deeks JJ, Dinnes J, D’Amico R, et al (2003) Evaluating non-randomised intervention studies. Health Technol Assess 7(27):8–10, 1–173 10.3310/hta7270 14499048

[CR9] DerSimonian R, Laird N (1986). Meta-analysis in clinical trials. Control Clin Trials.

[CR10] Duval S, Tweedie R (2000). Trim and fill: a simple funnel-plot–based method of testing and adjusting for publication bias in meta-analysis. Biometrics.

[CR11] Egger M, Smith GD, Schneider M, Minder C (1997). Bias in meta-analysis detected by a simple, graphical test. BMJ.

[CR12] Ellenberg JH (1994). Selection bias in observational and experimental studies. Stat Med.

[CR13] Frishman WH, Ky T, Ismail A (2001). Tobacco smoking, nicotine, and nicotine and non-nicotine replacement therapies. Heart Dis.

[CR14] Hedges LV, Olkin I (1985). Statistical methods for meta-analysis.

[CR15] Higgins JP, Thompson SG (2002). Quantifying heterogeneity in a meta-analysis. Stat Med.

[CR16] Higgins JP, Thompson SG, Deeks JJ, Altman DG (2003). Measuring inconsistency in meta-analyses. BMJ.

[CR17] Hong SN, Kim JH, Choe WH, Han HS, Sung IK, Park HS, Shim CS (2010). Prevalence and risk of colorectal neoplasms in asymptomatic, average-risk screenees 40 to 49 years of age. Gastrointest Endosc.

[CR18] Larsson SC, Orsini N, Wolk A (2005). Diabetes mellitus and risk of colorectal cancer: a meta-analysis. J Nat Cancer Inst.

[CR19] Lloyd-Jones D, Adams RJ, Brown TM (2010). Executive summary: heart disease and stroke statistics–2010 update—a report from the American Heart Association. Circulation.

[CR20] Neugut AI, Rosenberg DJ, Ahsan H, Jacobson JS, Wahid N, Hagan M, Rahman MI, Khan ZR, Chen L, Pablos-Mendez A, Shea S (1998). Association between coronary heart disease and cancers of the breast, prostate, and colon. Cancer Epidemiol Biomarkers Prev.

[CR21] Siegel R, Naishadham D, Jemal A (2012). Cancer statistics. CA Cancer J Clin.

[CR22] Stang A (2010). Critical evaluation of the Newcastle–Ottawa Scale for the assessment of the quality of nonrandomized studies in meta-analyses. Eur J Epidemiol.

[CR23] Sterne JA, Egger M (2001). Funnel plots for detecting bias in meta-analysis: guidelines on choice of axis. J Clin Epidemiol.

[CR24] Stone NJ, Robinson JG, Lichtenstein AH (2014). 2013 ACC/AHA guideline on the treatment of blood cholesterol to reduce atherosclerotic cardiovascular risk in adults: a report of the American College of Cardiology/American Heart Association Task Force on Practice Guidelines. J Am Coll Cardiol.

[CR25] Sutton AJ, Abrams KR, Jones DR, Sheldon TA, Song F (2000). Methods for meta-analysis in medical research.

[CR26] Tiong AY, Brieger D (2005). Inflammation and coronary artery disease. Am Heart J.

[CR27] Yang SY, Kim YS, Chung SJ, Song JH, Choi SY, Park MJ, Yim JY, Lim SH, Kim D, Kim CH, Kim JS, Song IS (2010). Association between colorectal adenoma and coronary atherosclerosis detected by CT coronary angiography in Korean men; a cross-sectional study. J Gastroenterol Hepatol.

[CR28] Zheng W, McLerran DF, Rolland B (2011). Association between body-mass index and risk of death in more than 1 million Asians. N Engl J Med.

